# The spatiotemporal dynamic evolution of post-stroke neuroinflammation: energy metabolism mechanisms of acute response and chronic progression

**DOI:** 10.3389/fphar.2026.1762764

**Published:** 2026-02-24

**Authors:** Yating Du, Yunfei Yang, Chunxiang Zhang, Qiong Yuan

**Affiliations:** Key Laboratory of Medical Electrophysiology, Ministry of Education & Medical Electrophysiological Key Laboratory of Sichuan Province, (Collaborative Innovation Center for Prevention of Cardiovascular Diseases), Institute of Cardiovascular Research, Southwest Medical University, Luzhou, China

**Keywords:** energy metabolism, glycolysis, immunity cells, ischemic stroke, lactic acid, neuroinflammation, stroke-induced immunosuppression syndrome

## Abstract

Ischemia stroke is a highly disabling and fatal disease in China. Neuroinflammation is the predominant pathological change in ischemic stroke. Various immune cells such as microglia and T cells are involved in the process of brain tissue damage during ischemic stroke; they play different roles during the acute and chronic phases, the disruption of which may directly affect the functional prognosis of stroke in animals and humans. Therefore, a deep understanding regarding the bidirectional regulatory mechanisms of neuroinflammation is essential for developing novel therapeutic strategies. In this review, we aim to introduce the recent research progress on neuroinflammation in the pathogenesis of ischemic stroke, while elucidating the energy metabolism changed induced immunity cells activation in ischemic stroke including glycolysis, lactic acid and succinate. We also review the RNA modification on energy metabolism changed. The information in this review provides valuable insights for further transitional research on stroke.

## 1 Introduction

Ischemic stroke, the most common subtype of stroke, is a primary cause of mortality and disability worldwide (Ntaios et al., 2020; Tu and Wang, 2023). It exhibits a highly complex and multi-stage neuro pathological cascade during its pathogenesis. Current clinical treatments mainly focus on relieving cerebral vascular occlusion through thrombolysis or thrombectomy; however, there are limited effective intervention strategies for the triggered neuroinflammatory response (Bourne et al., 2025). Although treatment strategies for ischemic stroke have been extensively explored, there are limited known safe and effective clinical treatment alternatives (Hankey, 2017), thus highlighting the need to explore new therapeutic targets (Alraddadi et al., 2025). Neuroinflammation is a key pathological feature of ischemic stroke, exhibiting a complex dual role during disease progression (Lombardozzi et al., 2026; Maida et al., 2020). During the acute phase (0–72 h), a neuroinflammatory cascade involving microglia, astrocytes, and peripherally infiltrating immune cells exacerbates neuronal damage and blood-brain barrier (BBB) disruption (Lu W. et al., 2023; Lu et al., 2024). Simultaneously, it triggers a strong innate immune response by releasing damage-associated molecular patterns (DAMPs) (Hernandez et al., 2023; Liu et al., 2021). However, during the subacute and chronic phases, moderate inflammatory responses can promote neural repair and functional remodeling (Lombardozzi et al., 2026). The disruption of this dynamic balance directly affects the functional prognosis of experimental stroke animals and patients with stroke (Lu W. et al., 2023; Lu et al., 2024). Therefore, a deep understanding regarding the bidirectional regulatory mechanisms of neuroinflammation is of great significance for developing novel therapeutic strategies (Lu et al., 2024; Zhang et al., 2021a). In this review, we aim to review the inflammation system activation and the mechanism in ischemia stroke impairment during recent 3–5 years.

## 2 Temporal characteristics of neuroinflammation after ischemic stroke

**FIGURE 1:**
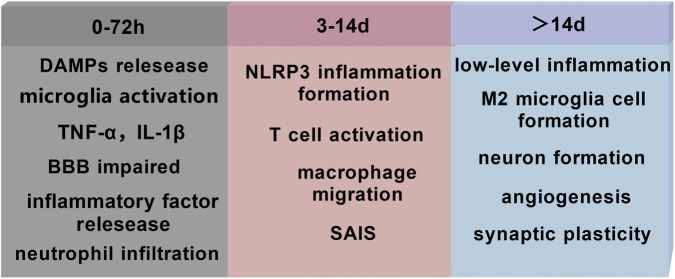
Characteristics of time-stage changes in neuroinflammation during ischemic stroke. The inflammation happened during different stages of ischemia stroke. The mainly changed in 0–72 h include DMAPs relesease; microglia activation; TNF-α and IL-1β; BBB impairment and neutrophil infiltration. During 3–14 days, mainly focus on NLRP3 inflammation formation, T cell activation, macrophage migration and SAIS. After 14 days, the brain maintain low-level inflammation, M2 microglia cell formation, neurons form regeneration, angiogenesis and synaptic plasticity. DAMPS, Damage-Associated Molecular Patterns TNF, Tumor necrosis factor; BBB, blood-brain barrier; NLRP3, NOD-like receptor protein 3; SAIS, stroke-associated immunosuppression.

Neuroinflammation after ischemic stroke is classified into the acute, subacute, and chronic phase. The acute phase (0–72 h) involves the release of danger signals and activation of innate immunity. Within the first 72 h after stroke, the neuroinflammatory response exhibits a burst-like feature. The neuroinflammatory cascade is activated as early as 36–133 min following stroke and progresses at an extremely rapid rate (Kowalski et al., 2023). This stage begins with the rapid release of DAMPs, triggering excessive microglia activation (Li et al., 2021b). Activated microglia overproduce pro-inflammatory factors such as tumor necrosis factor (TNF)-α, induced nitric oxidative stress (iNOS), and interleukin (IL)-1β (Li et al., 2021b), while inducing the breakdown of BBB (Lombardozzi et al., 2026). Neutrophils, as the main innate immune cells, rapidly infiltrate the ischemic area (Wang et al., 2025a); together with microglia, they constitute the core effector cell population of acute-phase neuroinflammation. This hyperinflammation exacerbates neuronal damage (Lombardozzi et al., 2026) and is closely associated with poor neurological functional prognosis (Lombardozzi et al., 2026). The subacute phase (3–14 days post-stroke) involves the initiation and regulation of adaptive immune responses. This phase marks the transition of the immune response from innate to adaptive immunity. The NOD-like receptor protein 3 (NLRP3) inflammasome continues to drive neuroinflammatory responses during this phase (Bellut et al., 2023), and delayed administration by even 1 day can improve long-term prognosis (Bellut et al., 2023). Meanwhile, peripheral macrophages begin to migrate across the BBB (Wang et al., 2025a), forming a complex interaction network with resident microglia. T-cell subsets begin to participate in inflammatory regulation (Wang et al., 2025a), but stroke-associated immunosuppression, which emerges during this time, may lead to systemic immunosuppression (Alraddadi et al., 2025). The inflammatory response in this phase has a distinct bidirectional regulatory feature: on one hand, it continuously produces pro-inflammatory mediators which exacerbate damage, and on the other hand, it initiates repair mechanisms (Lombardozzi et al., 2026). The chronic phase (>14 days) is characterized by a dynamic balance between persistent low-grade inflammation and nerve repair. There is a gradual increase in M2 polarization of microglia (Lombardozzi et al., 2026), promoting repair processes such as neurogenesis, angiogenesis, and synaptic plasticity (Lombardozzi et al., 2026). However, sustained activation of inflammasomes can still lead to long-term tissue damage (Bellut et al., 2023). Exosome-mediated intercellular communication becomes particularly important in this stage. For example, extracellular vesicles contain some inflammatory factors, such as CRP, MMP-9, CXCL4, PECAM1, IL-6, and OPN, which rapidly increase in patients with stroke 36–133 min after onset. This occurs earlier than the previously observed time of neuroinflammation in patients. Inflammatory regulation during the chronic phase requires a precise balance between pro-inflammatory and anti-inflammatory mechanisms (Alraddadi et al., 2025), and excessive suppression may hinder the necessary repair processes (Figure 1) (Lombardozzi et al., 2026).

## 3 Immunometabolic microenvironment characteristics following stroke

Following stroke, the ischemic region shows significant metabolic gradient differences; metabolomic studies have identified 4-guanidinobutyric acid (γ-guanidinobutyric acid) and glutamate two characteristics metabolisms to dived core infarct area and penumbra, which can provide new insights for clinical evaluation (Zhang et al., 2022b). Cerebral ischemia disrupts oxygen and energy supply, triggering a cascade of thyroid hormone metabolic stress responses (Huang et al., 2022; Lei et al., 2025). An energy metabolism crisis is characterized by acute compensatory enhancement of glycolysis, which induces pathological changes through enzymatic lipid peroxidation, driving early neuroinflammatory cascades (Huang Z. P. et al., 2023; Wang et al., 2025b). This metabolic disorder exhibits distinct spatiotemporal dynamic characteristics wherein energy depletion and hypoxia occur early in ischemia, activating resident glial cells and promoting peripheral immune cell infiltration into the brain parenchyma (Chen et al., 2021; Xie et al., 2023). A temporal association exists between the peak of immune cell infiltration and peak of metabolic stress, where the differentiation of T cell subsets is regulated by the local metabolic microenvironment (Chen et al., 2021); systemic immunosuppression then occurs as a secondary effect following the early stage of over-activation (Wu et al., 2022; Xie et al., 2023).

### 3.1 Glycolysis-oxidative phosphorylation transition and multi-immune cell activation

#### 3.1.1 Glycolysis induce phenotype switch of neutrophils

During the acute phase of ischemic stroke (24–72 h), neutrophils, as the first immune cells to infiltrate brain tissue, exhibit a pronounced N1 pro-inflammatory phenotype. This phenotype directly exacerbates brain tissue damage by releasing reactive oxygen species (ROS), protease, and pro-inflammatory cytokines such as IL-1β and TNF-α. Concurrently, neutrophils form neutrophil extracellular traps (NETs) within the brain parenchyma, and elevated levels of their plasma biomarkers are significantly associated with poor prognosis in stroke patients (Denorme et al., 2022). Furthermore, N1 phenotype neutrophils can obstruct cerebral capillaries, leading to impaired reperfusion and further expanding the ischemic penumbra damage. During the subacute phase of stroke (7–14 days), neutrophil phenotypes dynamically shift from N1 to the reparative N2 type. Experimental studies indicate that specific interventions, such as spinal cord stimulation (SCS), can promote neutrophil differentiation toward the N2 phenotype by inducing the IL-10/Stat3 signaling pathway (Gao Z. et al., 2025). N2 phenotype neutrophils exhibit pro-angiogenic and tissue-repair functions, characterized by upregulation of specific markers such as arginase-1 and CD206, and accelerate the progression of the inflammation resolution phase (Gao Z. et al., 2025; Richter et al., 2025). This phenotypic transition closely aligns with the time window for neurological functional recovery after stroke, confirming that neutrophils possess disease stage-dependent functional plasticity (Richter et al., 2025). Lactate, a key end product of glycolysis, accumulates significantly in the ischemic microenvironment and bidirectionally regulates neutrophil phenotypes through activation of the mammalian target of rapamycin complex 1 (mTORC1) signaling pathway (Dhanesha et al., 2022). Low concentrations of lactate promote the polarization of N2 repair phenotypes, enhancing their anti-inflammatory and tissue repair functions; whereas high concentrations of lactate drive neutrophils toward a pro-inflammatory N1 phenotype via mTORC1 activation, exacerbating inflammatory injury (Dhanesha et al., 2022). This concentration-dependent regulation reveals the dual role of lactate in the post-stroke immune microenvironment, where its accumulation level directly determines the inflammation/repair balance of neutrophils (Dhanesha et al., 2022). Inhibiting glycolysis with the glycolytic inhibitor 2-deoxy-D-glucose (2-DG) can reduce the polarization of microglia toward the pro-inflammatory M1 phenotype (Liu G. et al., 2023). In stroke models, selective inhibition of the glycolytic key enzyme hexokinase 2 (HK2) suppresses the pro-inflammatory activation of microglia, reduces oxidative stress and synaptic loss, and improves long-term functional outcomes.

#### 3.1.2 Molecular mechanisms of glycolytic reprogramming in regulating T cell subset differentiation

T cells undergo significant metabolic reprogramming during activation and differentiation, with enhanced glycolysis being a key characteristic. This metabolic shift not only provides energy and biosynthetic precursors for rapid T cell proliferation but also precisely regulates the differentiation fate and effector functions of different T cell subsets by influencing key signaling pathways and epigenetic modifications (Chen C. et al., 2022; Hochrein et al., 2022; Longo et al., 2025; Wu et al., 2023). In the neuroinflammatory environment following ischemic stroke, glycolytic reprogramming plays a particularly prominent role in regulating the differentiation of T-cell subsets, directly influencing disease progression. Studies indicate that the differentiation direction of CD4^+^ T cells is strictly controlled by glycolytic activity. Under neuroinflammatory conditions such as ischemic stroke, CD4^+^ T cells enhance glycolytic metabolism, which promotes the differentiation of pro-inflammatory Th17 cells while suppressing the generation of regulatory T cells (Treg). Increased glycolytic flux activates the mTORC1 signaling pathway and upregulates hypoxia-inducible factor-1α (HIF-1α), thereby stimulating the production of pro-inflammatory cytokines such as IL-17. This disrupts the Treg/Th17 balance and exacerbates neural damage (Hochrein et al., 2022). During the acute phase of ischemic stroke, glycolytic reprogramming drives pro-inflammatory T cells to infiltrate the ischemic brain parenchyma, directly exacerbating neural damage. Research indicates that the infiltration of CD8^+^ T cells into the brain parenchyma and their mediated neurotoxicity are key factors in the expansion of ischemic brain injury. Perioperative stroke can trigger metabolic abnormalities in peripheral CD8^+^ T cells, leading to the accumulation of succinate-2-hydroxyglutarate (S-2HG), which in turn enhances the neurotoxicity of CD8^+^ T cells and significantly aggravates ischemic brain injury and cognitive dysfunction (Zhang et al., 2022a). Furthermore, infiltrating T lymphocytes (including neutrophils and monocytes) disrupt microvascular integrity and secrete inflammatory factors such as IL-17A, increasing blood-brain barrier (BBB) permeability and promoting cerebral edema and hemorrhagic transformation. The glycolytic byproduct lactate activates mTORC1 signaling, which in turn paracrinely activates Th1 and Th17 cells, thereby amplifying neuroinflammatory responses (Meng et al., 2024).

Abnormal glycolytic metabolism in T cells during the chronic phase can establish a persistent ‘immunometabolic memory’ leading to long-term neurological deficits. Perioperative stroke-induced metabolic dysregulation in peripheral CD8^+^ T cells—such as the accumulation of S-2HG—not only alters T cell proliferation and differentiation but also persistently regulates immune-mediated brain injury, ultimately resulting in post-stroke cognitive impairment. This metabolic memory manifests as CD8^+^ T cell exhaustion: impaired glycolysis leads to reduced abilities in activation, differentiation, and proliferation, accompanied by decreased interferon-gamma (IFN-γ) secretion and a decline in the proportion of central memory CD8^+^ T cell subsets. At the molecular level, sustained lactate accumulation exacerbates brain damage by inducing neuronal death and activating A1-type astrocytes (Xiong et al., 2024). In the ischemic environment, myeloid-derived suppressor cells (MDSCs) differentiate into immunologically active myeloid cells. This process is accompanied by an increase in glycolysis levels, which impairs their immunosuppressive and neuroprotective functions, leading to persistent chronic inflammation (Hong et al., 2024; Zhu et al., 2025).

#### 3.1.3 Glycolysis induces phenotypic changes in microglia

**FIGURE 2:**
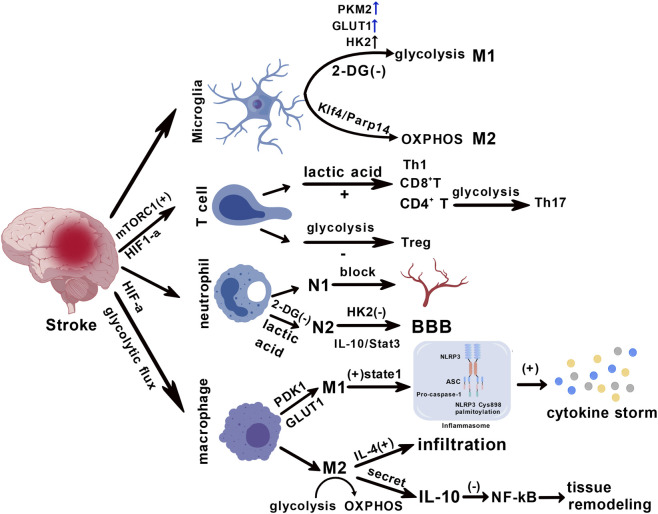
Metabolic reprogramming-mediated differentiation of multi-immunity cells. Microglia differentiate into the M1 phenotype through glycolytic metabolism, a process regulated by the upregulation of PKM2, GLUT, and HK2 proteins. They differentiate into the M2 phenotype via oxidative phosphorylation (OXPHOS) mediated by the Klf4/Parp14 signaling pathway. Lactic acid induces the activation of CD8^+^ T cells, CD4^+^ T cells, regulatory T cells (Treg), and T helper 1 (Th1) cells. Neutrophils can be activated to adopt the N1 and N2 phenotypes. Macrophages induced to the M1 phenotype contribute to cytokine storm formation, whereas those polarized to the M2 phenotype promote tissue remodeling.

The metabolic phenotype of microglia is significantly correlated with their functional polarization state in ischemic stroke (Yang et al., 2021). Activated pro-inflammatory (M1-type) microglia primarily rely on the glycolytic pathway for energy supply, characterized by increased glycolytic flux and substantial accumulation of lactate (Cheng et al., 2021; Yang et al., 2021). This glycolytic dominance directly amplifies the secretion of pro-inflammatory cytokines (such as TNF-α and IL-1β) by supplying biosynthetic precursors and modulating signaling molecules, thereby exacerbating neuroinflammatory damage (Cheng et al., 2021; Gao et al., 2025a; Yang et al., 2021). In contrast, neuroprotective anti-inflammatory (M2-type) microglia primarily rely on oxidative phosphorylation and fatty acid oxidation (FAO) to sustain their functions (Yang et al., 2021). Experimental evidence indicates that inhibiting glycolysis (for example, using 2-deoxyglucose) can effectively reduce pro-inflammatory polarization and improve neurological deficits (Figure 2) (Cheng et al., 2021; Luo et al., 2021; Won et al., 2025). This metabolic phenotype’s strong association with inflammatory polarization reveals the therapeutic potential of targeting the glycolytic pathway to regulate microglial function (Gao et al., 2025a; Yang et al., 2021). An ischemic and hypoxic environment prompts microglia to rapidly shift their energy metabolism pathways, transitioning from the basal metabolic state to a phenotype dominated by glycolysis (Cheng et al., 2021; Gao et al., 2025a; Ma et al., 2023; Wang et al., 2025b). This shift in metabolic characteristics not only impacts energy supply efficiency but also profoundly participates in the regulation of neuroinflammatory responses, constituting a critical link in the pathological progression following stroke.

#### 3.1.4 Glycolysis regulates macrophage differentiation

During the acute phase of ischemic brain injury, the polarization of macrophages/microglia toward the pro-inflammatory M1 phenotype is a core mechanism driving the exacerbation of neuroinflammation. The hypoxic microenvironment triggers metabolic reprogramming, shifting macrophages from oxidative phosphorylation to glycolytic metabolism, enabling rapid energy production to support their pro-inflammatory functions (Chen P. et al., 2025; Shen et al., 2024). Increased glycolytic flux upregulates hypoxia-inducible factor 1α (HIF-1α) and signal transducer and activator of transcription 1 (STAT1), which activates the NOD-like receptor thermal protein domain associated protein 3 (NLRP3) inflammasome. This subsequently induces pyroptosis and triggers the release of large amounts of pro-inflammatory cytokines (such as IL-1β and TNF-α), leading to the formation of a ‘cytokine storm’ (Shen et al., 2024). Furthermore, the upregulation of key glycolytic enzymes such as pyruvate dehydrogenase kinase 1 (PDK1) and glucose transporter 1 (GLUT1) sustains the metabolic foundation of the M1 phenotype (Chen D. et al., 2022; Shen et al., 2024). This metabolic-inflammatory positive feedback loop exacerbates brain tissue damage through reactive oxygen species (ROS) bursts and neuronal apoptosis pathways (Lu et al., 2025; Qiu et al., 2024). During the injury recovery phase, macrophages polarize toward the anti-inflammatory M2 phenotype, with their core functions relying on repair mechanisms mediated by specific effector molecules. Interleukin-10 (IL-10), a key anti-inflammatory cytokine secreted by M2 macrophages, alleviates neuroinflammation by suppressing the NF-κB pathway and promotes tissue remodeling (Wu et al., 2025). Simultaneously, the increased secretion of vascular endothelial growth factor (VEGF) improves local microcirculation and alleviates tissue hypoxia (Wu et al., 2025). At the metabolic level, M2 polarization requires a shift from glycolysis to oxidative phosphorylation (OXPHOS) (Cheng et al., 2026). This process is driven by cytokines such as IL-4 (Huang et al., 2023a). Studies indicate that IL-4 significantly enhances the infiltration of M2 macrophages in cerebral ischemic tissue and accelerates fibrotic scar formation by promoting the deposition of extracellular matrix proteins (e.g., fibronectin), thereby providing structural support for neural repair (Figure 2) (Huang et al., 2023a).

### 3.2 Immune regulatory effect of lactic acid accumulation and microenvironment acidification

Accelerated glycolysis can lead to substantial lactate production, causing local lactate accumulation and microenvironment acidification in brain tissue. Lactate is not merely a metabolic byproduct but also an important immunoregulatory molecule. On one hand, lactate can activate the mammalian target of rapamycin complex 1 (mTORC1) through autonomous signaling pathways, directly promoting the differentiation of myeloid-derived suppressor cells (MDSCs) (Zhou et al., 2024); on the other hand, lactate is also involved in epigenetic regulation—in ischemic brain tissue, the level of protein lactylation modification is significantly elevated, and non-histone lactylation affects the inflammatory process through molecular mechanisms that have not yet been clarified (Sun et al., 2023). In the peri-infarct zone, increased histone lactylation modification helps create an anti-inflammatory microenvironment and exerts neuroprotective effects (Lei et al., 2025). This lactate-mediated immunomodulation exhibits a dual nature: moderate lactylation can suppress neuroinflammation, whereas excessive lactate accumulation exacerbates microenvironment acidification, inhibits T-cell activity, and promotes the release of pro-inflammatory factors (Liang et al., 2025). Furthermore, lactate accumulation can regulate the NLRP3 signaling pathway through epigenetic mechanisms. In the ischemic microenvironment, lactate produced via lactate dehydrogenase-mediated processes can induce lactylation modification at the histone H3K18 site, altering the chromatin accessibility of inflammation-related genes (Yu et al., 2024). After inhibiting lactate dehydrogenase and reducing lactate levels, the activation capacity of the NLRP3 inflammasome can be restored (Li et al., 2022; Su et al., 2025). In cerebral ischemia models, lactate accumulation drives NLRP3-dependent pyroptosis by increasing histone lactylation levels, whereas blocking lactate production significantly attenuates inflammasome activation (Li et al., 2025b). This process can also form a positive feedback loop involving lactate and HIF-1α, further amplifying the immunosuppressive effect of the glycolysis-MDSCs axis (Huang T. et al., 2025; Zhang et al., 2025b).

The polarization state of microglia is closely associated with its core energy metabolic pathways. Pro-inflammatory (M1-type) microglia primarily rely on glycolysis to rapidly generate energy, characterized by the upregulation of key glycolytic enzymes (such as PKM2 and HK2) and glucose transporters (such as GLUT1), as well as increased lactate production (Fix et al., 2021; Hua et al., 2024). This glycolytic advantage promotes the synthesis and release of pro-inflammatory cytokines (such as IL-1β). In contrast, anti-inflammatory or reparative (M2-type) microglia primarily rely on oxidative phosphorylation (OXPHOS) and fatty acid oxidation to meet their energy demands, supporting their phagocytic clearance function and secretion of neuroprotective factors (Fix et al., 2021; Yang et al., 2021). Following stroke, microglia undergo a metabolic shift from OXPHOS to glycolysis, driving their polarization toward the pro-inflammatory M1 phenotype and exacerbating neuroinflammation (Peruzzotti-Jametti et al., 2021). Inhibiting glycolysis (e.g., using 2-Deoxy-D-glucose (2-DG) to inhibit HK2) can effectively reverse the pro-inflammatory polarization of microglia, reduce neuroinflammation, and improve neurological function (Liu Y. et al., 2023). Signaling pathways such as Klf5/Parp14 regulate the association between this metabolic and phenotypic transition (Simats and Liesz, 2022). As the earliest immune cells to infiltrate brain tissue following stroke, neutrophil activation is primarily dependent on glycolysis for energy supply. In the ischemic brain region, neutrophils rapidly produce ATP by upregulating glycolytic flux, supporting their functions such as chemotaxis, phagocytosis, and release of inflammatory mediators (Figure 2) (Hong et al., 2025). This glycolytic burst is a typical metabolic adaptation of neutrophils to hypoxic microenvironments and is directly associated with their pro-inflammatory phenotype (Hong et al., 2025). Effector T cells infiltrating the brain tissue (such as Th1 and CD8^+^ T cells) mainly rely on glycolysis to maintain their pro-inflammatory functions and rapid proliferative capacity (Figure 2) (Mohan and Kumar, 2025). In contrast, regulatory T cells (Tregs) tend to utilize OXPHOS and fatty acid oxidation, a metabolic pattern adapted to their immunosuppressive function (Figure 2) (Zhang et al., 2021b). Metabolic stresses (such as hypoxia and nutrient competition) in the brain microenvironment following stroke can significantly alter the balance of T cell subsets. Enhanced glycolytic flux promotes the differentiation of pro-inflammatory T cells and inhibits Treg function (Figure 2) (Op den Kamp et al., 2022). In addition, metabolic byproducts such as lactic acid can directly regulate the differentiation pathway of CD4^+^ T cells by inducing histone lactylation modification, thereby affecting the progression of neuroinflammation (Figure 2) (Op den Kamp et al., 2022). Dysregulation of T cell metabolic adaptability may lead to imbalanced immune responses following stroke, which in turn affects neural repair and the outcome of injury (Liu Y. et al., 2023; Xia et al., 2024b).

### 3.3 Succinate accumulation and succinylation modification

**FIGURE 3:**
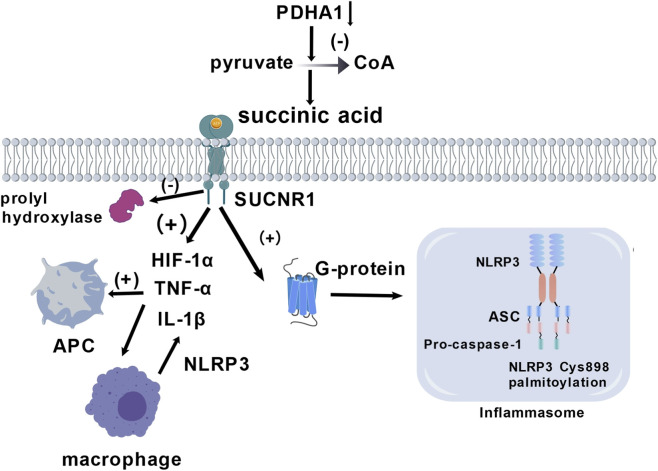
Succinic acid metabolism inducing inflammation activation. Succinic acid/SUCNR1 is the central role in APC, microgphage activation in different inflammation signal pathway including activating HIF-1α, NLRP3 inflammasome.

Metabolic reprogramming not only alters energy flow but also regulates inflammatory signaling through the metabolite-mediated post-translational modification of proteins. Succinate accumulates significantly in ischemic brain regions and activates pro-inflammatory signaling cascades by binding to the G protein-coupled receptor, SUCNR1 (Huo et al., 2021; Inamdar et al., 2023). The activation of SUCNR1 triggers the HIF-1α and TNF-α signaling pathways, thus promoting the inflammatory phenotype conversion of antigen-presenting cells (Inamdar et al., 2023). Succinate induces inflammation primarily via the following two signaling pathways: (1) Competitive inhibition of prolyl hydroxylase can stabilize HIF-1α and then upregulate IL - 1β transfection (Xia et al., 2022; Zhang et al., 2025); (2) activation of G protein-coupled signaling via the succinate receptor, SUCNR1, enhances NLRP3 inflammasome assembly and maturation-secretion of IL-1β (Liu K. et al., 2024). Clinical evidence suggests that serum and brain tissue succinate levels are significantly elevated in patients with stroke, and are positively correlate with the degree of inflammation (Liu K. et al., 2024; Xia et al., 2022). Targeting succinate metabolism (e.g., inhibiting succinate dehydrogenase) can effectively reduce IL-1β secretion by macrophages and alleviate neurological damage in experimental stroke models (Chang et al., 2024; Zhang et al., 2025a). This succinate-dependent metabolic reprogramming of macrophages constitutes a key molecular bridge connecting metabolic disorders with neuroinflammation (Liu K. et al., 2024; Stifel et al., 2022). In addition, succinate lowers the α-ketoglutarate/succinate ratio by inhibiting α-ketoglutarate-dependent dioxygenases (such as histone demethylase JMJD3), leading to DNA demethylation and enhanced transcription of pro-inflammatory genes (Cheng et al., 2022; Lee et al., 2022). In macrophages, succinate accumulation can induce IL-1β secretion, thus amplifying the neuroinflammatory cascade (Huo et al., 2021). In addition, succinylation modification is an important mechanism. Metabolic reprogramming affects the expression and activity of key enzymes, such as pyruvate dehydrogenase (PDHA1) (Figure 3). Under inflammatory stimulation, microglia often exhibit the downregulation of PDHA1 expression or impaired activity, thus hindering the conversion of pyruvate to acetyl-CoA, consequently affecting the homeostasis of tricarboxylic acid cycle intermediate products, including succinate (Gu et al., 2021). The accumulation of succinate promotes IL-1β secretion in macrophages and may exacerbate inflammatory responses by affecting the assembly or activation of the NLRP3 inflammasome (Figure 3).

## 4 RNA modification regulates the occurrence of neuroinflammation following stroke

### 4.1 The role of RNA methylation modification in neuroinflammation following stroke

Glycolysis, as an energy supply pathway rapidly activated after ischemia, undergoes metabolic reprogramming dynamically regulated by m6A RNA modification. Studies indicate that hypoxia-inducible factor HIF-1, a key regulator of glycolysis, may have its expression and function modulated by m6A modification. In animal models simulating ischemic stroke, dynamic changes in m6A modification show significant spatiotemporal correlation with energy metabolism disorders. For instance, the middle cerebral artery occlusion (MCAO) model in rats reveals upregulated expression of the methyltransferase METTL14 in microglia post-ischemia, with its variation trend synchronizing with the progression of ischemic injury (Xu et al., 2022). Meanwhile, both the MCAO model and the oxygen-glucose deprivation/reoxygenation (OGD/R) cell model confirmed that m6A modification levels are significantly elevated in neurons within the ischemic penumbra. Furthermore, the m6A reader protein IGF2BP1, which is enriched in this region, recognizes energy metabolism-related RNAs, suggesting that m6A modifications exhibit spatial specificity in the metabolically compensatory zone surrounding the ischemic core (Lei et al., 2025). Single-cell sequencing studies further revealed that 12 h after transient MCAO in mice, the expression of energy metabolism-related genes in cortical astrocytes was significantly altered, and the m6A modification levels of these genes were closely associated with the process of cellular metabolic reprogramming (Ma et al., 2022). Furthermore, ischemia-reperfusion (I/R) injury can trigger rapid dynamic changes in m6A modifications within brain tissue, with a time course that closely aligns with mitochondrial oxidative phosphorylation dysfunction and adenosine triphosphate (ATP) depletion (Xia et al., 2024a; Yang et al., 2025).

During the progression of ischemic stroke, oxidative stress, impaired energy metabolism, and m6A modification form a tightly interconnected feedback regulatory loop. Following cerebral ischemia-reperfusion, the surge of reactive oxygen species (ROS) and reactive nitrogen species (RNS) rapidly alters the activity of m6A methyltransferases (such as METTL3) and demethylases (such as ALKBH5), leading to abnormal m6A modifications in energy metabolism-related genes (e.g., genes encoding glycolytic enzymes and mitochondrial respiratory chain complexes) (Rabolli et al., 2025; Yang et al., 2025). This aberrant modification further regulates the translation efficiency of target mRNAs through reader proteins such as YTHDF1, exacerbating glycolytic pathway disruption and mitochondrial dysfunction, thereby creating an energy crisis (Li L. et al., 2023; Xu et al., 2025). Notably, energy metabolism collapse (such as ATP depletion) can reciprocally activate the m6A methylation process through the AMPK signaling pathway, forming a positive feedback loop of ‘oxidative stress → dysregulated energy metabolism → abnormal m6A modification → further metabolic deterioration’ (Chen F. et al., 2022; Rabolli et al., 2025). For instance, resolvin D1 (RvD1) can break this vicious cycle by activating AMPK to promote glutamine metabolic reprogramming in microglia, enhancing oxidative phosphorylation to maintain ATP supply, while simultaneously suppressing m6A-mediated stabilization of inflammatory factor mRNAs (Li L. et al., 2023).

### 4.2 Double-edged sword effect of METTL3/METTL14 complex in ischemia-reperfusion injury

The METTL3/METTL14 complex exhibits a complex double-edged sword effect in the pathological process of stroke. In ischemia-reperfusion injury models, METTL3 exhibits neuroprotective effects, and its overexpression can alleviate brain damage caused by insufficient cerebral blood flow (Chauhan and Kaundal, 2023). However, this complex simultaneously upregulates inflammation-related targets by mediating RNA hypermethylation: RNA sequencing and m6A-eCLIP experiments confirm that METTL3-mediated RNA hypermethylation can upregulate the transcription level of NLRP1 while downregulating that of KLF4, and these changes are mediated by YTHDF1 and YTHDF2 (Chien et al., 2021). In spinal cord ischemia-reperfusion injury, METTL3, as a key RNA methyltransferase, may regulate NLRP3 inflammasome activation through m6A modification, thereby exacerbating inflammatory responses and neuronal death (Guan et al., 2025). METTL14 expression is significantly upregulated in microglia in an ischemic stroke model (Li Y. et al., 2023), indicating its potential role in regulating neuroinflammation.

### 4.3 Regulation of neuroprotective pathways by FTO/ALKBH5 demethylase

Fat mass and obesity-associated protein (FTO), as an m6A demethylase, exhibits a clear neuroprotective function in acute ischemic stroke. Animal experiments have shown that adeno-associated virus 9-mediated FTO overexpression can significantly reduce post-stroke m6A hypermethylation levels in the brain tissue; alleviate damage to gray matter and white matter; and improve motor function recovery, cognitive ability, and depression-like behavior (Chokkalla et al., 2023; Zhang et al., 2024), and this protective effect is gender-independent (Crockett et al., 2021). At the molecular level, FTO regulates the maturation process of miR-320-3p by inhibiting the m6A methylation of pri-miR-320, thereby affecting the m6A methylation level of SLC7A11 mRNA, and ultimately inhibiting oxidative stress and ferroptosis induced by oxygen-glucose deprivation/reoxygenation in neurons (Peng et al., 2024). In addition, FTO and ALKBH5 together constitute a demethylase system. Clinical sample analysis also confirms that FTO expression is downregulated in the brain tissue of patients with stroke (Ye et al., 2023).

### 4.4 Reading the protein YTHDF1/2/3-mediated inflammatory signal selective recognition

**FIGURE 4:**
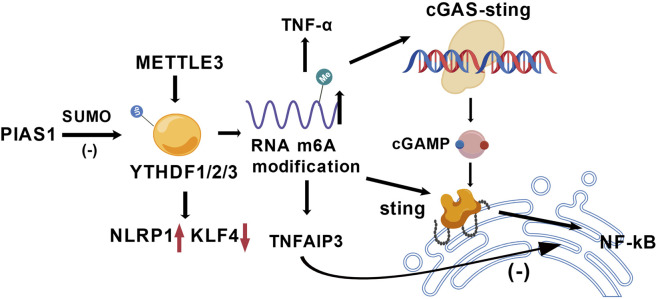
The function of YTHDF1/2/3 on mRNA m6A modification after stroke. PIAS1 is SUMO-modified and then inhibits YTHDF1/2/3 expression. YTHDF1/2/3 is also regulated by METTL3, which mediates RNA m6A modification. After RNA m6A modification, TNF-α is secreted and stimulates the cGAS-STING signaling pathway, which results in NF-κB protein activation. Additionally, YTHDF1/2/3 promote the upregulation of NLRP1 protein expression and the downregulation of KLF4 protein.

YTHDF family proteins (YTHDF1/2/3), as m6A reader proteins, regulate inflammatory signaling by selectively recognizing methylated modifications. In ischemic stroke models, YTHDF proteins exhibit spatiotemporal specificity in their expression. Single-cell sequencing studies reveal that astrocytes undergo significant alterations in energy metabolism-related genes within 12 h post-ischemia (Lu K. J. et al., 2023; Ma et al., 2022). Studies indicate that reduced YTHDF1 expression is closely associated with disturbances in neuronal energy metabolism and exacerbated inflammatory responses (Liu et al., 2021). Spatially, YTHDF1 is predominantly highly expressed in neurons and participates in regulating mitochondrial function (Ke et al., 2023), whereas YTHDF2 is enriched in infiltrating immune cells and influences neuroinflammation by modulating lipid metabolism-related genes (He et al., 2024; Wei et al., 2024). This spatiotemporal heterogeneity in expression and distribution suggests that YTHDF family members may play distinct regulatory roles across different stages of stroke and in various cell types. YTHDF1 and YTHDF2 act as downstream effector molecules of METTL3, which are involved in the post-transcriptional regulatory process of mediating NLRP1 upregulation and KLF4 downregulation (Figure 4) (Chien et al., 2021). The function of YTHDF proteins is regulated by post-translational modifications. PIAS1-mediated SUMOylation can restrict the activity of YTHDF1 and YTHDF3, thereby affecting their ability to degrade RNA (Zak et al., 2024).

## 5 Peripheral-central immune interaction mechanisms

**FIGURE 5:**
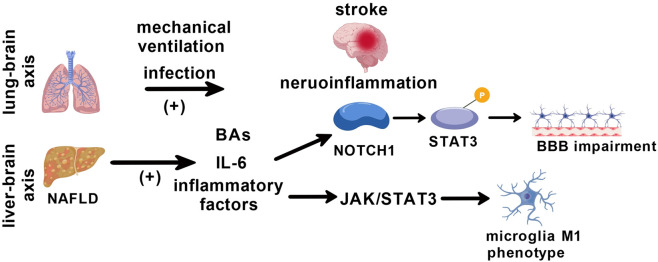
Interaction between peripherol organs and brian inflammation. Schematic diagram illustrating the neuro-immunoinflammatory mechanisms. Post-stroke, NAFLD exacerbates systemic inflammation. Inflammatory factors (e.g., IL-6) disrupt blood-brain barrier (BBB) integrity via astrocyte NOTCH1/STAT3 signaling and drive microglial polarization toward a pro-inflammatory M1 phenotype via the JAK/STAT3 pathway, amplifying central nervous system damage. This axis represents a key pathway in remote organ communication following stroke.

As an important peripheral immune organ, the spleen plays a key role in the brain-spleen axis communication following stroke, participating in the interactive regulation between the central and peripheral immune systems through multiple mechanisms (Wang et al., 2025c; Yu et al., 2021). Post-stroke neuroinflammation can trigger immune responses in multiple organ systems, making peripheral organs a ‘second battlefield’ for immune reactions (Wu et al., 2022). The brain-lung axis is an important remote organ communication pathway. Pulmonary infection and mechanical ventilation following stroke can significantly worsen the prognosis of ischemic stroke (Xie et al., 2024). Stroke-induced bacterial colonization in the lungs is associated with gut microbiota dysregulation, which cannot be prevented using anti-inflammatory treatment (Diaz-Marugan et al., 2023). Stroke increases the hepatic production of primary bile acids but reduces their production in the gut. Systemic immune activation and immunosuppressive state triggered after a stroke can lead to the dysfunction of remote organs, thereby forming a vicious cycle which results in an adverse prognosis (Wu et al., 2022). The liver-brain axis, as a key pathway connecting hepatic dysfunction with central nervous system diseases, plays a central role in the process by which non-alcoholic fatty liver disease (NAFLD) promotes ischemic stroke through the underlying neuro-immunoinflammatory mechanisms (Petta et al., 2016; Tuttolomondo et al., 2018b; Zanoli et al., 2019). Systemic inflammatory factors and metabolic products affect the integrity of the BBB, glial cell activation, and neuronal function through this axis, forming a cascade amplification effect. Systemic inflammation induced by NAFLD directly damages the BBB structure through the liver-brain axis. Clinical evidence suggests that elevated blood levels of inflammatory factors (such as IL-6) in patients with NAFLD activate the NOTCH1 signaling pathway of astrocytes, promote STAT3 phosphorylation, upregulate the secretion of pro-permeability factors, thus leading to increased BBB permeability (Figure 5) (Maida et al., 2020; Mora et al., 2024). After crossing the BBB, hepatic inflammatory factors directly regulate the phenotypic transformation of microglia. In the state of NAFLD, microglia polarize toward the pro-inflammatory M1 phenotype, a mechanism associated with the sustained activation of the JAK/STAT3 signaling pathway (Chen S. et al., 2025; Tuttolomondo et al., 2018a).

## 6 Therapeutic strategies and translational research

The phase-specific characteristics of post-stroke neuroinflammation provide an important basis for precise treatment. The focus of treatment during the subacute phase should shift to regulating adaptive immune responses, particularly by modulating the infiltration of peripheral immune cells through the chemokine network (Wang et al., 2024). In the chronic phase, it is necessary to balance pro-inflammatory and anti-inflammatory responses, targeting the regulatory role of T cell subsets in persistent low-grade inflammation (Wang et al., 2024; Yuan et al., 2021). Combined therapeutic strategies have become an innovative trend in post-stroke neuroinflammation intervention. On one hand, nanocarriers can simultaneously deliver multiple chemotherapeutic drugs to the target site, achieving multi-target synergistic therapy (Ghosh et al., 2021). On the other hand, the combined use of immune checkpoint inhibitors and immunomodulators can potentially enhance anti-inflammatory effects (Beattie et al., 2021; Granot-Matok et al., 2023).

### 6.1 Anakinra translational medicine significance of neuroprotective agents

Anakinra is a recombinant human interleukin-1 receptor antagonist (rhIL-1Ra) with a structure highly similar to that of the natural endogenous IL-1Ra. As an anti-inflammatory cytokine, anakinra competitively blocks the signaling of both IL-1α and IL-1β by specifically binding to the interleukin-1 type I receptor (IL-1R1) (Chaparro-Cabanillas et al., 2025; Huang et al., 2025a). Its molecular mechanism relies on binding affinity for IL-1R1 but does not recruit the receptor accessory protein (IL-1RAcP), thereby failing to activate downstream inflammatory signaling pathways (Diep et al., 2022). This structural characteristic makes it a natural inhibitor of the IL-1 signaling pathway, playing a key regulatory role in pathological processes such as tumor formation and neuroinflammation (Diep et al., 2022). Anakinra exerts neuroprotective effects through anti-inflammatory therapy: as an interleukin-1 receptor antagonist, it directly inhibits the IL-1β-driven inflammatory cascade. Experimental studies confirm that it reduces the release of pro-inflammatory factors and the infiltration of inflammatory cells in ischemic brain tissue, significantly alleviating neuroinflammation, particularly in reperfusion models (Maunier et al., 2022; Venugopal et al., 2021).

### 6.2 Fingolimod inhibits lymphocyte migration and regulates inflammatory factors

Fingolimod, as a non-selective S1P receptor modulator, binds to all subtypes of S1P receptors except for S1PR2 (Basnet et al., 2025; Liu et al., 2025). The phosphorylated active form FTY720-P induces the internalization of S1PR1 and downregulates cell surface receptor expression, thereby blocking the S1P-S1PR1 signaling axis (Bravo et al., 2022; Liu et al., 2025). Fingolimod antagonizes S1P function, thereby inhibiting the activation of pro-inflammatory signaling pathways such as the NLRP3 inflammasome, NF-κB, and MAPKs (Basnet et al., 2025), and reducing the expression of pro-inflammatory cytokines including tumor necrosis factor-α (TNF-α) and interleukin-1β (IL-1β) (Basnet et al., 2025). Furthermore, studies suggest that Fingolimod may increase the number of regulatory T cells (Tregs) in ischemic brain regions, mediating immunosuppressive effects through FoxP3+ cells and further enhancing neuroprotective functions (Malone et al., 2021). Fingolimod demonstrates multi-target advantages in the neuroprotective treatment of ischemic stroke due to its unique ability to modulate S1P receptors, with a well-established mechanistic basis particularly in immune regulation and blood-brain barrier protection.

## 7 Prospective

A major challenge currently faced in research is the significant differences between humans and animal models in the temporal progression of neuroinflammation. Clinical observations have found that the neuroinflammatory cascade is activated within 36–133 min following a stroke, a speed far exceeding previous understanding (Kowalski et al., 2023). This shows a marked difference from the temporal characteristics observed in rodent animal models, which may lead to a mismatch in the time window when therapeutic strategies developed based on animal models are translated to clinical practice. Notably, inflammatory markers post-stroke in humans rise rapidly within the first 2 h and continue to increase for 24 h, suggesting a dynamic change during the hyperacute phase which is difficult to fully replicate in existing animal models. This difference in time scales suggests that we need to establish a more precise time correspondence between human and animal models to optimize the selection of therapeutic intervention time windows (Castillo-Gonzalez et al., 2024). Preclinical studies have shown that microglia, as key effector cells of neuroinflammation, exhibit spatiotemporal dynamic changes in their activation and polarization states with alterations in the microenvironment (Kwon, 2022). Such changes may be regulated by age-related immune senescence and sex hormone levels, but further elaboration on the relevant mechanisms is still desired (Lu et al., 2024).
